# A Website to Promote Physical Activity in People With Type 2 Diabetes Living in Remote or Rural Locations: Feasibility Pilot Randomized Controlled Trial

**DOI:** 10.2196/diabetes.6669

**Published:** 2017-10-19

**Authors:** Jenni Connelly, Alison Kirk, Judith Masthoff, Sandra MacRury

**Affiliations:** 1 Physiology, Exercise and Nutrition Research Group Faculty of Health Science and Sport University of Stirling Stirling United Kingdom; 2 Physical Activity and Health Research Group School of Psychological Sciences and Health University Of Strathclyde Glasgow United Kingdom; 3 University of Aberdeen Aberdeen United Kingdom; 4 Department of Diabetes and Cardiovascular Science University of Highlands and Islands Inverness United Kingdom

**Keywords:** blood glucose, diabetes, physical activity, rural, virtual trainer, Web based

## Abstract

**Background:**

Research supports the use of Web-based interventions to promote physical activity in diabetes management. However, previous interventions have found poor levels of engagement or have not included health professionals and people with diabetes in the design of the tool.

**Objective:**

To develop and explore the feasibility and indicative effect of a Web-based physical activity promotion intervention in people diagnosed with type 2 diabetes living in remote or rural locations.

**Methods:**

A qualitative approach using focus groups that included patients with diabetes and health professionals were run to identify key concepts, ideas, and features, which resulted in the design of a physical activity website. This site was tested using a quantitative approach with a qualitative 6-month pilot study that adopted a three-armed approach. Participants were randomized into three groups: a control group who received written diabetes-specific physical activity advice; an information Web group, a Web-based group who received the information online; and an intervention Web group, an interactive Web-based group who received online information plus interactive features, such as an activity log, personalized advice, and goal setting.

**Results:**

A website was designed based on patient and health professional ideas for effective physical activity promotion. This website was tested with 31 participants, 61% (19/31) male, who were randomized into the groups. Website log-ins decreased over time: 4.5 times in month 1, falling to 3 times in month 6. Both the information Web group—mean 134.6 (SD 123.9) to mean 154.9 (SD 144.2) min—and the control group—mean 118.9 (SD 103.8) to mean 126.1 (SD 93.4) min, *d*=0.07—increased time spent in moderate-to-vigorous physical activity, but this decreased in the intervention Web group—mean 131.9 (SD 126.2) to mean 116.8 (SD 107.4) min.

**Conclusions:**

Access to online diabetes-specific physical information was effective in promoting physical activity in people with type 2 diabetes; access to interactive features was not associated with increases in activity.

**Trial Registration:**

International Standard Randomised Controlled Trial Number (ISRCTN): 96266587; http://www.isrctn.com/ISRCTN96266587 (Archived by WebCite at http://www.webcitation.org/6tzX6YesZ)

## Introduction

Regular physical activity has been shown to be beneficial in the management of type 2 diabetes [1-4], with guidelines recommending that adults should accumulate 150 minutes of moderate physical activity a week [5,6]. Up to 80% of people with type 2 diabetes do not meet these recommendations [7], highlighting the need for the development of effective interventions.

Physical activity interventions have been delivered through face-to-face contact, telephone contact, print and mail materials, and group-based activities [8,9]. Although many interventions have been effective in stimulating physical activity behavior changes, implementation of these methods of delivery into current diabetes practice has often been restricted by lack of time and appropriate personnel for effective delivery. It is also important to identify and create channels of information delivery that can reach a large and broad range of the diabetes population, including those who may not or cannot access more traditional methods of delivery, for example, those living in remote or rural locations [10].

Innovations in technology and access to the Internet have led to an increased number of technology-based interventions. Using technology to deliver an intervention offers several advantages, including the potential reach, continuing availability, and cost containment of the intervention [11]. Web-based interventions have been successfully implemented in promoting physical activity in diabetes self-management [12]. However, these interventions often resulted in poor levels of user engagement [13] and frequently reported major decreases in usage over time [14,15]. There is therefore a need to develop a tool with specific features that aim to increase user engagement.

The primary aim of this study was to utilize coproduction methodology to develop a Web-based physical activity promotion intervention. The secondary aims were to evaluate the feasibility and indication of effectiveness of using this intervention to promote physical activity in people with type 2 diabetes living in remote and rural localities.

## Methods

### Study Design

This study utilized a mixed-methods research design to incorporate coproduction into the development of the intervention. Using a qualitative approach, focus groups were conducted with people living with type 2 diabetes to explore the key features of a Web-based physical activity promotion intervention. Quantitative data was collected on overall and individual component use of the intervention and an objective measure of physical activity and sedentary behavior was conducted. The full study was approved by the North of Scotland research ethics committee (12/NS/0115) and was conducted according to the principles of the Helsinki agreement. The trial was registered with the International Standard Randomised Controlled Trial Number (ISRCTN) Registry (ISRCTN96266587).

### Research Design of Focus Groups

#### Recruitment

Participants were recruited through purposive sampling from diabetes clinics, diabetes volunteer lists, and posters put up in general practice surgeries. Inclusion criteria included the following: diagnosis of type 2 diabetes, over 18 years of age, and ability to communicate verbally in English.

A total of 30 participants (18 males, 12 females) with a mean age of 63 (SD 12) years participated in four patient focus groups—focus group 1 (n=8), focus group 2 (n=8), focus group 3 (n=7), and focus group 4 (n=7)—held over a 4-month period. One group was also run with health professionals (n=6). Participants who attended these groups lived in rural or remote locations in Scotland.

#### Data Collection and Analysis

NVivo (QSR International) and thematic analysis were used with initial codes generated in a systematic fashion across the dataset. Coding was carried out and collated into tables, which were separated by themes and subthemes. This was then reviewed to ensure the themes worked in terms of each individual code. Coding checks were conducted on a subset of three of the five transcripts by researchers external to the research team to ensure consistency within themes as recommended by Barbour [16].

### Research Design for Website Testing

#### Overview

To test the effectiveness of the intervention, in addition to the active intervention group there were two comparator groups: one group had access to the website, but not interactive features to control for any effect of online information access, and a second was provided with written information only.

#### Recruitment and Randomization

A total of 31 participants, not previously involved in the focus groups, who were diagnosed with type 2 diabetes were recruited from primary and secondary care sites from three rural localities in Highland Region, Scotland. The aim was to recruit between 10 and 12 participants per group. The method of randomization for this pilot trial was to use opaque envelopes to conceal group allocation from the researcher, which the Cochrane Review reports as having a low risk of bias [17]. Envelopes were prepared by a researcher who was not involved in the study and allowed for equal numbers across the recruitment areas in each of the intervention groups.

Participants were included in the study if they met the following criteria: diagnosed with type 2 diabetes managed through lifestyle or oral medication; over 18 years old; resident in Inverness, Isle of Skye, and Sutherland areas; and had access to a computer with Internet access. Exclusion criteria included the following: treatment with insulin therapy, to reduce the potential for immediate effects on glycemic control and need for insulin adjustment; unable to understand study requirements or give informed consent; visual or hearing impairments or physical disability; or diabetes-related complication that precluded ability to increase physical activity.

Participants met with the research nurse on three occasions: baseline, 3 months, and 6 months. During the first visit, demographic and medical details were collected and participants were randomized using opaque envelopes into one of the three groups: the interactive Web group (InterG) had online access to diabetes-specific physical activity information and interactive features; the information Web group (InfoG) were given online access to diabetes-specific physical activity information, but not interactive features; and the control group received leaflets based on the website material. Each of the three sites was inducted into the study in a stepped-wedge approach over 6 weeks.

### Primary Outcome: Website Measures

To assess use of the website, log-on to the site was monitored with each participant given personalized log-on details, which were then used to measure contact over the 6 months. Access to the site without logging in was not measured. The use of interactive features on the site was recorded for each participant to assess frequency of use and thus determine those features most likely to be useful in future versions of the website.

### Secondary Outcomes

#### Assessment of Physical Activity

To assess the effectiveness of the intervention, data on physical activity were collected. Data were also collected on standard anthropometric measurements and glycated hemoglobin (HbA1c), a measure of glycemic control, both of which may be impacted by change in physical activity. These data were collected from all groups at three intervals across the duration of the study. Participants wore an ActiGraph GT3X+ monitor (ActiGraph, LLC) around the waist for 7 days at baseline, 3 months, and 6 months. Accelerometer data were downloaded and analyzed using ActiLife data analysis software, version 6.10 (ActiGraph, LLC). A 60-second epoch was applied with a minimum wear time of 10 hours per day on at least 4 days, including one weekend day. A time of 60 minutes and over of consecutive zeroes was considered nonwear time and was excluded from analysis. The following Freedson adult cut-points were applied to categorize physical activity [18]: sedentary, 0-99 cpm; light, 100-759 cpm; lifestyle, 760-1951 cpm; moderate, 1952-5724 cpm; vigorous, 5725-9498 cpm; and very vigorous, ≥9499 cpm.

#### Anthropometric Measurements and Blood Sampling

All measures were carried out by a research nurse. Body mass index (BMI) was calculated as weight in kg over height in m^2^(kg/m^2^). Waist circumference was calculated in cm and was taken in the midpoint between the iliac crest and the lowest rib. A blood sample was drawn at baseline, 3 months, and 6 months and analyzed at the hospital clinical laboratory for HbA1c using a high performance liquid chromatography (HPLC) method (Tosoh Bioscience) on diabetes control and complications-aligned equipment.

### Statistical Analysis

Data were analyzed using SPSS version 22 (IBM Corp). After normality-testing, repeated-measures analysis of variance (ANOVA) or the nonparametric Friedman k related-samples tests were used, Bonferroni corrections were applied. Data are presented as mean (SD). As this was a pilot study, a sample size calculation was not performed so, in addition to the arbitrary significance level of .05, the effect size is reported. Effect size was calculated using Cohen *d*, where 0.2 indicates a small effect, 0.5 indicates a medium effect, and 0.8 indicates a large effect. Outliers more than 3 standard deviations from the mean were removed per variable.

## Results

### Web Features Developed in Response to the Focus Groups

#### Overview

Excerpts from focus groups are displayed in Table 1 and Multimedia Appendix 1.

#### Virtual Coach

Groups highlighted a perceived need for support to increase their physical activity (Excerpt 1.0). To accomplish this, the website was centered on a virtual coach, Dave; all interface conversations with the site were between the user and Dave. This was to ensure that people felt that the website provided personal advice structured to each individual.

#### Ask the Expert

The need for support led to the creation of an *Ask the expert* section of the website to allow users the opportunity to speak to the physical activity expert. This allowed users the chance to ask physical activity-related questions related to their diabetes to a physical activity expert through the interface of Dave.

#### Physical Activity Tracker

A tool for users to evaluate the physical activity they had undertaken was suggested to enable users to make more active choices (Excerpt 2.0). A tracker diary to help monitor activity was built into the site. This tracker allowed users to enter type and duration of the activity displayed in a bar chart permitting users to see days they were active and days they were not active (Excerpt 2.1). A range of physical activity options were available on the drop-down list, based on the American College of Sports Medicine list of activities [19]. The website remembered the activities entered; if more than one activity was carried out in the one day, the activities were stacked.

**Table 1:** Quotes from focus groups used to design key features of the website.

Subthemes from focus groups	Example excerpt highlighting subtheme meaning	Participant type	Excerpt number
Importance of support	“I need help in exactly what I need to do; it’s not the advice of what to do it’s the support of doing it.”	Patient	1.0
Monitoring physical activity and diabetes			
	“...sitting down and thinking about what I’ve done, when I’ve done it and if I haven’t done it I think it would make me think about why I’m not being active.”	Patient	2.0
	“...by displaying your activity, say in graph form, it would give you some sort of target and to help you evaluate the days you weren’t active.”	Patient	2.1
Methods to increase user engagement	“... if you do lands’ end and do it in bite-size chunks it doesn’t matter how long it takes you to do it, you will still have done it at the end.”	Health professional	3.0

#### Goals and Challenges

An online physical activity consultation based on the transtheoretical model [20] was developed allowing each user to set up realistic incremental goals based on current behavior. The consultation was conducted through a Web interface and gave personalized advice based on what was entered.

Any activity entered into the site was converted into walking using metabolic equivalent values [19] and the distance was added into a challenge (Excerpt 3.0). Users could pick a challenge to complete over a few weeks or months and in theory complete a hypothetical marathon through any number of activities emphasizing the spectrum of activity at a level to suit each individual.

#### Activities in Local Areas

A Google map was created of physical activity opportunities in Highland Region, pinpointed so users could zoom into their area and find out what was available. This was developed through contact with the Highland council and local groups to ensure opportunities on the map were current. All pinpoints on the map had contact details or Web links to the person or facility running the activity.

### Online Intervention

Baseline characteristics of the groups are described in Table 2. Statistical tests—one-way ANOVA (continuous data), chi-square, or Fisher’s exact tests (categorical data)—indicated a significant difference between groups for weight: *F*_2,28_=3.6, *P*<.04. This was accounted for by the higher percentage of males (80%) in the InfoG; when looking at BMI there were no significant differences across the group. Mean duration of diabetes was highest in the InfoG and lowest in the InterG: 9.3 (SD 5.5) years versus 5.7 (SD 1.6) years.

**Figure 1:**
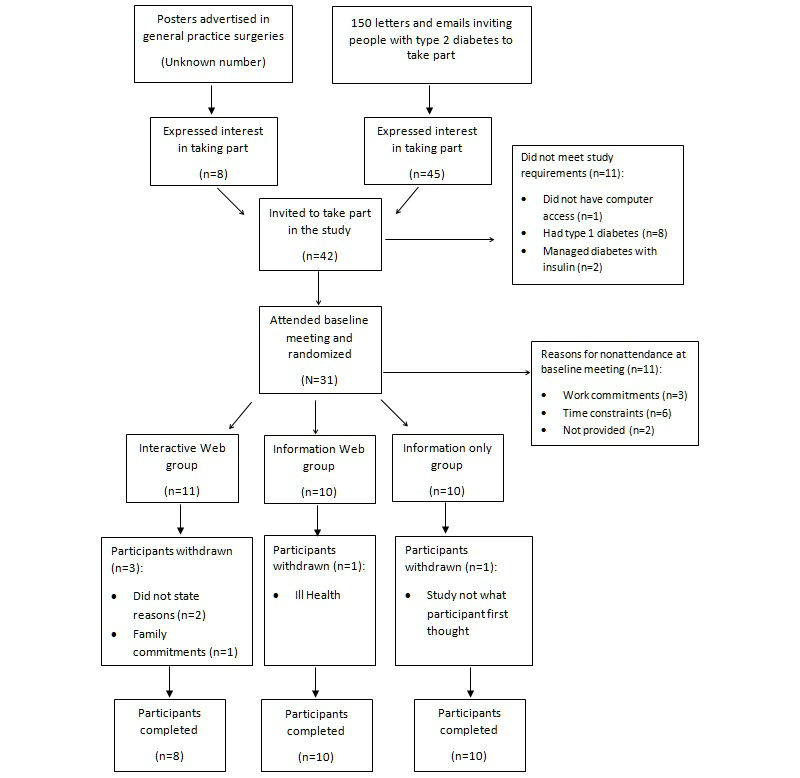
Recruitment and attrition flow diagram for pilot randomized controlled trial.

Regarding study attrition, 5 participants out of 31 (16%) withdrew from the study: 3 out of 11 (27%) InterG, 1 out of 10 (10%) InfoG, and 1 out of 10 (10%) control group. Figure 1 shows the flow diagram of recruitment and attrition.

### Changes in Primary Outcome: Website Measures

Total log-in counts for the website was 262. Over the first 3 months, each participant logged in an average of 12.5 (SD 15.7) times dropping to 11.3 (SD 37.1) times from 3- to 6-month follow-up. There was a large range in the number of log-ins, starting at zero and going up to 50 times in 1 month. In the last 2 months, only 1 person continued to use the website. Table 3 highlights log-in rates per month of study.

**Figure 2:**
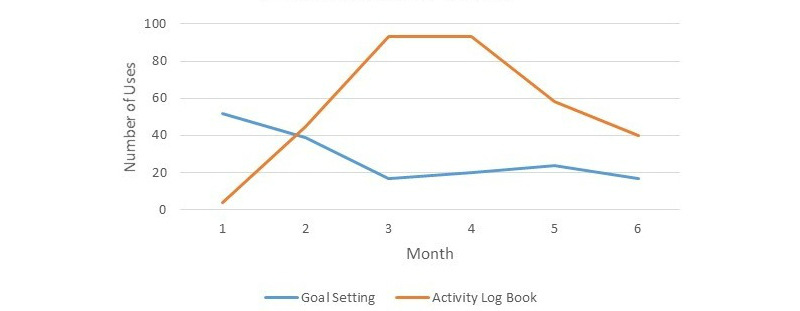
Use of interactive features over time.

Out of the features, only *goal setting* and *log book* were used. The *log book* feature was used 142 times in the first 3 months and increased to 191 times in the second 3 months. The *goal setting* feature was used 108 times in the first 3 months and dropped to 61 times in the second 3 months. Usage broken down per month is highlighted in Figure 2. The most common goal was walking, with 41 goals set in the first 3 months and 83 goals set in the second 3 months. Other goals included swimming, stair climbing, cleaning, cycling, gardening, and circuit training.

### Changes in Secondary Outcomes

#### Physical Activity

Table 4 reports the ActiGraph accelerometer-defined physical activity results broken down into groups at baseline, 3 months, and 6 months.

**Table 2:** Baseline demographic characteristics of participants in the interactive Web, information Web, and control groups.

Characteristic	Interactive Web group (n=11)	Information Web group (n=10)	Control group (n=10)
Gender (male), n (%)	6 (55)	8 (80)	4 (40)
Age (years), mean (SD)	67.3 (10.4)	66.2 (8.4)	66.5 (6.0)
Duration of diabetes (years), mean (SD)	5.7 (1.6)	9.3 (5.5)	6.9 (4.1)
Weight (kg), mean (SD)	83.1 (11.6)	100.0 (15.9)	88.7 (16.1)
BMI^a^(kg/m^2^), mean (SD)	30.3 (4.2)	33.0 (5.5)	31.4 (5.8)
Metformin therapy, n (%)	9 (82)	5 (50)	6 (60)
Lifestyle only, n (%)	2 (18)	5 (50)	4 (40)

^a^BMI: body mass index.

**Table 3:** Log-in rates per month broken down into intervention months.

Month	Number of log-ins			
	Minimum, n	Maximum, n	Sum, n	Mean (SD)
1	0	14	48	4.4 (4.2)
2	0	27	55	5.0 (7.7)
3	0	16	34	3.1 (4.9)
4	0	39	40	3.6 (11.7)
5	0	50	50	4.5 (15.1)
6	0	34	34	3.1 (10.3)

**Table 4:** Changes in ActiGraph accelerometer-defined physical activity data broken down into groups and collection dates.

Physical activity	Interactive Web group (n=11), mean (SD)			Information Web group (n=10), mean (SD)			Control group (n=10), mean (SD)		
	Month 0	Month 3	Month 6	Month 0	Month 3	Month 6	Month 0	Month 3	Month 6
Light (min/wk)	1136.8 (334.7)	896.8 (176.4)	1160.4 (302.2)	1169.9 (501.0)	1225.2 (422.0)	1211.6 (376.0)	1271.2 (288.3)	951.8 (280.8)	1249 (313.1)
Lifestyle (min/wk)	360.5 (182.9)	294.5 (145.7)	332.8 (302.2)	301.4 (191.2)	397.9 (188.9)	361.6 (188.9)	374.9 (174.3)	303.8 (56.3)	459.3 (149.8)
Moderate (min/wk)	127.1 (127.0)	72.2 (64.1)	108.4 (108.3)	127.5 (178.0)	134.0 (147.8)	140.1 (40.9)	112.9 (102.7)	83.4 (66.8)	121.7 (96.7)
Vigorous (min/wk)	4.8 (3.9)	2.0 (1.7)	8.4 (5.9)	7.1 (2.1)	8.5 (2.7)	14.8 (4.6)	5.7 (1.4)	2.5 (0.8)	9.6 (1.5)
Moderate-to-vigorous (min/wk)	131.9 (126.2)	74.2 (65.6)	116.8 (107.4)	134.6 (123.9)	142.5 (135.9)	154.9 (144.2)	118.9 (103.8)	86.5 (74.1)	126.1 ( 93.4)
Step count (steps/wk)	33,571 (20,134)	22,949 (10,398)	30,058 (16,116)	33,655 (24,684)	35,271 (20,679)	37,083 (25,387)	33,412 (20,845)	25,716 (10,414)	34,597 (17,675)
Sedentary time (total min/wk)	3275 (646)	2509 (756)	3004 (485)	3202 (759)	3129 (997)	3055 (807)	2993 (662)	2429 (776)	2833 (764)
Sedentary time (max bout in min)	72.5 (16.8)	41.4 (35.4)	37.7 (38.4)	69.5 (17.4)	78.8 (21.6)	55.4 (7.4)	68.2 (22.6)	47.4 (25.8)	47.6 (35.8)
Sedentary time (mean bout in min)	22.4 (1.2)	22.4 (2.3)	22.8 (3.9)	22.8 (3.0)	23.5 (2.6)	22.8 (4.1)	21.6 (2.8)	21.6 (2.9)	21.6 (2.6)

#### Moderate-to-Vigorous Physical Activity

Regarding moderate-to-vigorous physical activity (MVPA), the InterG dropped from 131.9 (SD 126.2) to 74.2 (SD 65.6) min/week at 3 months, increasing to 116.8 (SD 107.4) min/week at 6 months: *d*=-0.12. The control group dropped from 118.9 (SD 103.8) to 86.5 (SD 74.1) min/week at 3 months, increasing to 126.1 (SD 93.4) min/week at 6 months: *d*=0.07. The InfoG increased from 134.6 (SD 123.9) to 142.5 (SD 135.9) min/week at 3 months, furthering increasing to 154.9 (SD 144.2) min/week at 6 months: *d*=0.15.

#### Total Sedentary Time

The InterG’s sedentary time decreased from 3275 (SD 646) to 2509 (SD 756) min/week at 3 months, increasing to 3004 (SD 485) min/week at 6 months: *d*=0.5. In the InfoG, sedentary time decreased from 3202 (SD 759) to 3129 (SD 997) min/week at 3 months, further decreasing to 3055 (SD 807) min/week at 6 months: *d*=0.18. Within the control group, there was a decrease in total sedentary time from 2993 (SD 662) to 2429 (SD 776) min/week at 3 months, followed by an increase to 2833 (SD 764) min/week at 6 months: *d*=0.2. Table 5 reports the ActiGraph accelerometer-defined physical activity results broken down into groups at baseline, 3 months, and 6 months.

The InterG increased in light intensity percentage from 23.2 (SD 4.9) at baseline to 24.0 (SD 6.1) at 3 months: *d*=0.1. They also increased in lifestyle intensity percentage from 7.3 (SD 2.5) at baseline to 7.8 (SD 2.8) at 3 months: *d*=0.18. Sedentary behavior percentage decreased slightly in the InterG group from 66.7 (SD 7.6) at baseline to 66.5 (SD 6.1) at 3 months: *d*=0.02.

The control group increased their sedentary behavior percentage from 62.9 (SD 6.3) at baseline to 64.4 (SD 10.4) at 3 months: *d*=0.17.

**Table 5:** ActiGraph accelerometer-defined physical activity data broken down into wear-time percentage.

Physical activity	Interactive Web group (n=11), mean (SD)			Information Web group (n=10), mean (SD)			Control group (n=10), mean (SD)		
	Month 0	Month 3	Month 6	Month 0	Month 3	Month 6	Month 0	Month 3	Month 6
Light (%/wk)	23.2 (4.9)	24.0 (6.1)	25.0 (4.6)	24.3 (4.1)	25.0 (3.4)	25.0 (4.0)	26.7 (4.5)	25.2 (6.3)	26.7 (7.4)
Lifestyle (%/wk)	7.3 (2.5)	7.8 (2.8)	7.2 (2.3)	6.3 (3.0)	8.1 (3.6)	7.6 (3.0)	7.8 (3.5)	7.6 (3.3)	9.8 (5.1)
Moderate-to-vigorous (%/wk)	2.7 (2.2)	1.9 (2.3)	2.2 (3.0)	2.8 (3.0)	3.0 (1.9)	3.5 (2.8)	2.5 (1.9)	2.3 (2.5)	2.7 (2.3)
Sedentary time (total %/wk)	66.7 (7.6)	66.5 (6.1)	65.6 (6.1)	66.6 (7.5)	63.0 (6.4)	62.8 (7.2)	62.9 (6.3)	64.4 (10.4)	60.7 (11.9)

**Table 6:** Changes in physiological measures broken down into groups and collection dates.

Physiological measure	Interactive Web group (n=11)			Information Web group (n=10)			Control group (n=10)		
	Month 0	Month 3	Month 6	Month 0	Month 3	Month 6	Month 0	Month 3	Month 6
Weight (kg), mean (SD)	83.1 (11.6)	84.5 (13.1)	81.8 (12.8)	100.0 (15.9)	100.1 (14.4)	99.0 (15.8)	88.7 (16.1)	87.1 (15.7)	87.7 (5.6)
BMI^a^(kg/m^2^), mean (SD)	30.3 (4.2)	30.5 (4.0)	29.3 (4.1)	33.0 (5.5)	33.0 (5.1)	32.7 (5.6)	31.4 (5.8)	30.9 (5.8)	31.0 (5.6)
Waist circumference (cm), mean (SD)	104.0 (10.9)	106.0 (10.4)	102.8 (10.2)	118.3 (12.7)	113.6 (10.3)	114.2 (11.1)	108.3 (13.4)	107.2 (14.4)	101.0 (11.7)
HbA1c^b^(mmol/mol), mean (SD)	57.7 (11.2)	56.7 (10.0)	54.1 (9.5)	51.8 (8.0)	55.7 (7.6)	57.5 (9.1)	55.3 (13.7)	54.4 (15.6)	50.5 (5.9)
HbA1c, %	7.5	7.4	7.1	6.9	7.3	7.5	7.2	7.1	6.8

^a^BMI: body mass index.

^b^HbA1c: glycated hemoglobin.

#### Changes in Physiological Measures

All changes in physiological data are displayed in Table 6. Waist circumference decreased in the InfoG from 118.3 (SD 12.7) to 113.6 (SD 10.3) cm at 3 months, then increased to 114.2 (SD 11.1) cm at 6 months: *d*=0.34. It decreased in the control group from 108.3 (SD 13.4) cm at baseline to 107.2 (SD 14.4) cm at 3 months, then further decreased to 101.0 (SD 11.7) cm at 6 months: χ^2^_9_= -2.1, *P*<.02, *d*=0.5.

The InterG HbA1c decreased from 57.7 (SD 11.2) to 56.7 (SD 10.0) mmol/mol at 3 months, further decreasing to 54.1 (SD 9.5) mmol/mol at 6 months: *d*=0.34. The InfoG increased from 52.4 (SD 8.2) to 55.8 (SD 8.0) mmol/mol at 3 months, then decreased to 55.0 (SD 4.7) mmol/mol at 6 months: *d*=-0.38. The control group dropped from 55.3 (SD 13.7) to 54.4 (SD 15.6) mmol/mol at 3 months, further decreasing to 50.5 (SD 5.9) mmol/mol at 6 months: *d*=0.45.

## Discussion

### Principal Findings

This composite study is unique in that it reports on patient-identified features of a Web-based physical activity promotion intervention, overall and individual component use of the online intervention, together with change in physical activity. Patient-identified features included a *physical activity tracker*, *user support*, *goal setting*, *ask the expert*, *what is on*, and *interactive challenges*. Of the identified and included features within the online intervention, only the *activity log book* and *goal setting* were used.

Overall access to the website was good, specifically in the first 3 months of the intervention. This reduced in the second half of the intervention, which is common in Web-based interventions [13,21]. However, when education was combined with interactive elements, it did not result in any significant changes in physical activity. The two interactive features that were consistently used were *goal setting* and the *physical activity log book*; of these, neither appeared to be particularly effective in increasing physical activity, in contrast to previous research where those who used goal setting and log books had greater increases in physical activity [11].

Including patients in the design was key in the development of the current intervention. Even though the International Organization for Standardization principles [22]—recognized to ensure quality management—were followed, with more time, user-design workshops would have been helpful. These workshops would allow those using the site to test the features they deemed to be useful in an iterative fashion in process evaluation to determine their role in promoting activity. Longer-term interventions should be conducted to assess sustainability and strategies to increase engagement with the site.

This study provides some support for the use of online diabetes education in the promotion of physical activity. Although no significant change was reported in physical activity levels, a trend toward increasing physical activity was recorded in the InfoG, with 50% (5/10) in the InfoG meeting the current guidelines for physical activity at the end of the study. Online, tailored, physical activity advice has been shown to be effective in the general population [23], with interactive emails resulting in greater increases in physical activity. These are encouraging findings and endorse access to specific Web-based information to increase time spent in physical activity. Access to diabetes-specific physical activity information should be considered in endeavors to support patients with type 2 diabetes in becoming more physically active.

Access to interactive features resulted in a nonsignificant drop in physical activity. The reason for this is unclear, but this pattern was mirrored in the control group and seasonal reasons described in other studies could be postulated to explain the pattern [24]. However, a stepped-wedge method was used for recruitment into the trial, with equal numbers of participants randomized into each group per site and each site starting the intervention at a separate time. Given that these results were not observed in the InfoG makes it less likely and raises the question of whether issues with the interactive part of the website may have been a factor.

There was no significant difference in wear time across all groups and time points and all participants met wear-time criteria defined in the methods section. There were discrepancies in the data for the InterG in terms of light physical activity, lifestyle physical activity, and sedentary time, as well as for the control group in sedentary time from baseline to 3 months. However, none of these were significant and had low effect sizes. The main ActiGraph secondary outcome was MVPA and there was no difference in weekly wear-time percentage compared to minutes.

The current intervention did not contain any specific information on decreasing sedentary behavior; however, there was a trend toward decreases in total time spent in sedentary behavior, which may have been at the expense of increasing physical activity, which concurrently decreased. Thereafter, as physical activity increased, total sedentary time subsequently also increased in parallel, possibly due to a compensatory increment in resting time as individuals became more active. The benefits of decreasing sedentary spells are becoming more widely studied, with improvements in metabolic health suggested [25] and an acknowledgment that decreasing sedentary time is just as important as increasing physical activity in terms of health outcomes [26]. Moreover, evidence has shown that even people who meet the current guidelines for physical activity suffer adverse effects from too much sitting time, irrespective of meeting physical activity guidelines [27].

Although the study was not powered to detect significant changes and the mean HbA1c level reflected reasonable control at baseline, for the majority of patients in the InterG there were nonsignificant decreases in HbA1c across the 6 months. This may reflect the shorter duration of diabetes and higher percentage of participants receiving oral antidiabetic therapy in this group. The explanation for the upward trend in HbA1c with increases in MVPA in the InfoG is not clear, although it has been reported in other studies [28]. Possible reasons may include changes in diet or medication associated with increased physical activity, a component that was not a measured outcome of this study.

Waist circumference was used as a surrogate marker for abdominal fat mass; significant reductions were observed in the InfoG despite a lack of change in weight. Studies have shown that even without weight loss, increased physical activity is associated with reductions in fat mass [29], which can improve insulin sensitivity and in turn lead to improvement in blood glucose levels.

### Limitations

The study had a small sample size with only 31 participants in total. As this was a pilot intervention, no sample size calculation was undertaken and effect size was reported as an alternative. Future research should include in-depth follow-up, such as qualitative interviews, to explore the issues participants may or may not have had with the interactive features and so participants can provide feedback on what was useful and/or effective in promoting activity and engagement with the site.

All groups received new information on top of usual care procedures. The information received was diabetes-specific physical activity advice to aid in the promotion of physical activity. This new information, as well as increased patient contact, could influence outcomes.

### Comparison With Prior Work

Unlike previous work, this paper reports on the development and feasibility testing of the codesigned tool. It reports on what health professionals and people with type 2 diabetes considered to be essential tools for engagement with the site and support to increase physical activity and compared this with what was actually used. This study supported the use of a Web-based physical activity promotional intervention to communicate personalized physical activity education, which resulted in increased physical activity behavior in people with type 2 diabetes.

### Conclusions

Web-based physical activity information was associated with a trend toward increased physical activity across a 6-month intervention in people with type 2 diabetes. Interactive features were not effective in increasing physical activity participation.

## Appendix

Multimedia Appendix 1 Excerpts from focus groups that aided in the development of the website.

## Abbreviations

ANOVA: analysis of variance
BMI: body mass index
HbA1c: glycated hemoglobin
HPLC: high performance liquid chromatography
InfoG: information Web group
InterG: interactive Web group
ISRCTN: International Standard Randomised Controlled Trial Number
MVPA: moderate-to-vigorous physical activity
