# Supplementary material for: A Website to Promote Physical Activity in People With Type 2 Diabetes Living in Remote or Rural Locations: Feasibility Pilot Randomized Controlled Trial
Source: JMIR Diabetes. 2017 Oct 19;2(2):e26. doi: 10.2196/diabetes.6669 (PMC6238854; doi:10.2196/diabetes.6669)
Supplement: Multimedia Appendix 1 [file diabetes_v2i2e26_app1.pdf]

**Focus Group results**		
**Theme- Important website information**		
**Sub Themes from within the major theme;**	**Example excerpt highlighting sub theme meaning**	**Participant Type**
**The role of physical activity in diabetes management**	*'we don't really get advice on exercise, yeah usually it's all about the eating, we have diabetes, it's all about the eating'*	Patient
	'we're told that we should become more active, that it benefits our diabetes but were not told why or how'	Patient
	'I like to think of myself as quite active as there are so many benefits to activity not just for diabetes so I think it's important that my patients know these benefits not only for their diabetes but for their overall health'	Health Professional
**The benefits of physical activity to diabetes**	*'we already know it's supposedly good for us, we know it reduces blood pressure and is good for your heart'*	Patient
	'just stating that exercise reduces blood sugar isn't enough, I want to know why it's going to'	Patient
**Complications and physical activity**	*'are there any exercises that are really good to lower my glucose, what will lower it for quicker and for longer'*	Patient
**Theme- Key Features of a physical activity website**		
**Sub Themes from within the major theme;**	**Example excerpt highlighting sub theme meaning**	**Participant Type**
**Monitoring Physical activity and diabetes**	*'I have doubts about how much physical activity people do, I think I am quite active, so being able to monitor it somewhere would be useful'*	Patient
	sitting down and thinking about what I've done, when I've done it and if I haven't done it I think it would make me think about why I'm not being active'	Patient
	'by displaying your activity say in graph form, it would give you some sort of target and to help you evaluate the days you weren't active'	Patient
	'you're only cheating on yourself if you cheat, you have to be honest, it's for you so you need to be honest with it'	Patient

**Setting goals and targets to increase activity**	*'it has to be tailored to the specific individual need of each users in order for it to work, it can't be generic I wouldn't use it, I need structured advice that's relevant to me'*	Patient
	'there has to be the option to help set goals make them incremental, so they can start smaller and work their way up'	Patient
**Local facility awareness**	*'people like us living in rural areas are less likely to have access to leisure facilities like they have in big cities'*	Patient
	'they need physical activity information that is relevant to them in their local area'	Health Professional
	'if somebody clicked on Oban and found that there's a walking group daily that would be great to go along to'	Patient
**Theme- Support**		
**Sub Themes from within the major theme;**	**Example excerpt highlighting sub theme meaning**	**Participant Type**
**Importance of Support**	*'I need help in exactly what I need to do; it's not the advice of what to do it's the support of doing it'*	Patient
**The supportive role**	*'a trainer that's going to give me the diabetes physical activity expertise I need that I won't get anywhere else'*	Patient
	'a trainer that's going to give me the diabetes physical activity expertise I need that I won't get anywhere else'	Patient
**Theme-User Engagement**		
**Sub Themes from within the major theme;**	**Example excerpt highlighting sub theme meaning**	**Participant Type**
**Methods to increase user engagement**	*'monitoring physical activity would be a good motivation, it would allow me to evaluate what I have done as well as what I still need to'*	Patient
	'if you do lands' end and do it in bite size chunks it doesn't matter how long it takes you to do it, you will still have done it at the end'	Health Professional
